# Toxic Effects of BPAF on Marine Medaka (*Oryzias melastigma*) During Embryo–Larval Stages

**DOI:** 10.3390/toxics13090773

**Published:** 2025-09-12

**Authors:** Jiahao Gao, Tianyang Zhou, Zuchun Chen, Ning Zhang, Yusong Guo, Zhongduo Wang, Wenjun Shi, Zhongdian Dong

**Affiliations:** 1 Key Laboratory of Aquaculture in South China Sea for Aquatic Economic Animal of Guangdong Higher Education Institutes, College of Fishery, Guangdong Ocean University, Zhanjiang 524088, China; jhgao2001@163.com (J.G.); zhuxin_2025@foxmail.com (T.Z.); zcchen1999@163.com (Z.C.);; 2 Guangdong Provincial Key Laboratory of Pathogenic Biology and Epidemiology for Aquatic Economic Animals, College of Fishery, Guangdong Ocean University, Zhanjiang 524088, China; 3 Guangdong Provincial Key Laboratory of Chemical Pollution and Environmental Safety & MOE Key Laboratory of Theoretical Chemistry of Environment, SCNU Environmental Research Institute, South China Normal University, Guangzhou 510006, China; wenjun.shi@m.scnu.edu.cn

**Keywords:** Bisphenol AF, *Oryzias melastigma*, embryotoxicity, behavioral, gene expression

## Abstract

BPAF (Bisphenol AF), one of the primary substitutes for BPA (Bisphenol A), is widely used in the production of plastics, optical fibers, and other materials. During the use of these products, BPAF inevitably enters the environment and exerts toxic effects on animal growth, development, reproduction, immunity, neurology, and genetics. This study employed marine medaka (*Oryzias melastigma*) as the experimental model to evaluate the toxicological impacts of BPAF on early development. Embryos were exposed to four BPAF concentrations (0, 1 μg/L, 10 μg/L, and 100 μg/L) for 14 days (embryonic to larval stages), followed by phenotypic measurements, behavioral analysis, and gene expression detection. The results demonstrated that BPAF exposure induced developmental malformations and reduced survival rates in marine medaka embryos, with embryo survival negatively correlated with BPAF concentrations. Additionally, BPAF significantly decreased embryonic heart rates, and the 100 μg/L BPAF group exhibited prolonged embryo hatching time and reduced hatching success. In newly hatched larvae, BPAF exposure led to decreased body length, reduced heart rates, and significant suppression of swimming activity, characterized by increased resting time and reduced swimming distance. BPAF exposure altered the expression levels of genes associated with cardiovascular function (e.g., *tbx2b*, *arnt2*), the HPT axis (e.g., *tg*, *dio3a*, *trh*, *trhr2*, *tpo*), and neurodevelopment (e.g., *ache*, *elavl3*, *gfap*) in the medaka larvae. These transcriptional perturbations are proposed as potential molecular mechanisms underlying the observed phenotypic effects, including reduced heart rates and suppressed swimming behavior in the study. Molecularly, BPAF exposure significantly disrupted the expression of genes related to the cardiovascular system, HPT axis, and nervous system.

## 1. Introduction

BPAF (Bisphenol AF) is a primary substitute for BPA (Bisphenol A) and shares similar applications in plastic manufacturing, food packaging, optical fibers, and medical devices. With increasing restrictions or bans on BPA in multiple regions and countries, the production and consumption of BPAF have risen significantly. Unlike BPA, whose migration limit has been tightened to 0.05 mg/kg under EU Regulation (EU) 2018/213, its fluorinated substitute BPAF is currently neither included in the REACH Annex Restrictions List nor subject to a limit value in EU food contact material regulations. However, concerns about BPAF’s hazards are growing. The EU plans to designate BPAF and its salts as Substances of Very High Concern (SVHC) by August 2025. In the United States, annual BPAF production ranged from 4.5 to 226 tons between 1986 and 2002 [1], while China produced approximately 1500 tons in 2015 (ECHA 2015). The current annual total production of BPAF in China remains unknown, but a manufacturing plant in Jiaxing City, China, produced over 100 tons of BPAF in 2009 alone [2]. As early as 2012, research indicated that China had approximately 40 BPAF production bases [3]. However, this surge in BPAF usage has also led to its widespread environmental contamination. Song et al. detected BPAF in nearly half of 52 sewage samples from 30 Chinese cities, with a maximum concentration of 45.1 ng/g [4]. Zhang et al. analyzed 16 BPs in 20 Chinese water treatment plants, reporting BPAF concentrations ranging from not detected (ND) to 10.8 ng/L in source water and ND-4.7 ng/L in drinking water [5]. A survey conducted by the U.S. Environmental Protection Agency on 74 national public wastewater treatment plants across 35 U.S. states revealed that BPAF was detectable in 46% of sewage sludge samples, with concentrations ranging from <1.79 to 72.2 ng/g [6]. Environmental residues have also been identified in rivers, sediments, soil, indoor dust, and well water near BPAF manufacturing facilities in China [2]. BPAF can enter marine environments via riverine transport or direct wastewater discharge. Global reports of BPAF in marine environments, including seawater, sediments, and fish tissues, highlight its persistence and bioaccumulation potential, with some coastal waters reaching μg/L concentrations [7,8]. Water and sediment samples collected from the Tagus River along the northeastern Atlantic coast showed BPAF concentrations ranging from 0.025 to 40 ng/mL and 1.25 to 200 ng/g [7], respectively. BPAF concentrations in marine sediments collected from the Ebro Delta in Spain were <LOD-0.6 ng/g. Additionally, BPAF was detected in fish plasma (<LOD-6.7 ng/mL), liver (<LOD-0.17 ng/g), and muscle (<LOD-11.6 ng/g) [8]. These increasing concentrations of BPAF in aquatic systems pose significant threats to aquatic organisms, particularly fish. In this context highlight the critical need for systematic monitoring and ecological risk assessments of BPAF in marine ecosystems.

Compared to BPA, BPAF exhibits significantly higher bioaccumulation capacity and environmental persistence [9,10,11], as well as stronger estrogenic activity, which adversely impacts estrogen-sensitive organs such as the uterus, mammary glands, and testes [12,13], thereby posing greater ecological and health risks. In adult zebrafish (*Danio rerio*), BPAF exposure induces reproductive toxicity, leading to structural lesions in ovaries and testes, and impaired germ cell development [14]. It also triggers thyroid toxicity by altering thyroid hormone-related gene transcription, resulting in thyroid follicular epithelial hyperplasia, hypertrophy, and colloid depletion [14,15,16]. Chronic exposure of adult zebrafish to 10 μg/L BPAF for 28 days revealed sex-specific transcriptional responses: males exhibited more differentially expressed genes enriched in spermatogenesis-related pathways, whereas females showed enrichment in circadian regulation of gene expression. Both sexes displayed hepatic histopathological alterations and suppressed antioxidant capacity [17]. Prolonged 120-day exposure to 5, 25, 125 μg/L BPAF (from embryonic to adult stages) disrupted plasma sex hormone levels, dysregulated HPG axis-related genes in the brain, liver, and gonads, and reduced fertilization rates with increased teratogenicity in offspring [18]. In marine medaka (*Oryzias melastigma*) females exposed to BPAF for 120 days, swimming speed initially increased but declined at higher concentrations, while 367.0 μg/L BPAF significantly elevated body weight and condition factor, alongside delayed oocyte maturation at medium-to-high concentrations [19]. Additionally, BPAF causes neuroendocrine disruption, characterized by elevated cortisol levels and suppressed adrenal hormone production [15]. Huang et al. demonstrated that BPAF reduces survival and hatching rates, disrupts cardiovascular development in marine medaka embryos [20]. These findings collectively emphasize the multi-systemic toxicity of BPAF across species and life stages, but current toxicity studies of BPAF are focused on freshwater and lack attention to early developmental stages in fish, requiring urgent ecological risk assessment.

Salinity—a critical environmental factor influencing pollutant toxicity to fish [21,22]—is often overlooked in current studies. Current toxicity studies of BPAF have mostly focused on zebrafish, yet these data inadequately reflect its ecological risks in marine ecosystems due to distinct physiological and environmental adaptations between freshwater and marine species. Thus, employing marine fish models is essential for accurate risk assessment of BPAF in marine environments. This species offers distinct advantages, including high reproductive rates, broad salinity tolerance, and sensitivity to environmental pollutants [23,24]. The marine medaka is an ideal model organism for studying BPAF’s effects in marine ecosystems. These traits enable robust experimental designs for evaluating BPAF’s multisystem toxicity under environmentally realistic salinity regimes.

Using marine medaka embryos, we conducted a 14-day exposure study to evaluate the developmental and growth-related effects of BPAF at concentrations of 0, 1, 10, and 100 μg/L. Key parameters such as larval swimming behavior and gene expression related to cardiovascular, neuroendocrine, and thyroid systems were assessed. This study aims to provide crucial insights into BPAF’s developmental toxicity during the embryo-to-larval transition, contributing to a more accurate risk assessment of BPAF in marine environments and informing future water quality regulations.

## 2. Materials and Methods

### 2.1. Chemicals, Fish Maintenance and Chemical Exposure

BPAF (CAS 1478–61–1, purity ≥ 98%) and dimethyl sulfoxide (DMSO, purity ≥ 99.5%) were purchased from Sigma–Aldrich (St. Louis, MO, USA). BPAF was dissolved in DMSO to prepare the stock solution, which was stored at −20 °C for experimental use.

Marine medaka used in this study were obtained from the South China Sea Key Laboratory of Aquatic Economic Animal Propagation & Breeding, Guangdong Ocean University, and maintained in a recirculating artificial seawater system under controlled conditions: temperature 26 ± 1.0 °C, salinity 30 ± 1, and a 14 h:10 h light—dark cycle. Fertilized eggs were collected directly from females in the culture system, and unfertilized/dead embryos were removed under a stereomicroscope. Viable embryos were transferred to Petri dishes containing artificial seawater (salinity 30) for exposure experiments. Based on reported BPAF concentrations in surface waters [2], three exposure concentrations (1, 10, and 100 μg/L) were selected. The initial BPAF stock solution used was 10 mg/mL. This was then diluted twice in a stepwise manner to obtain exposure stock solutions of 1 mg/mL and 0.1 mg/mL. At the start of the experiment, 5 μL of BPAF stock solution was added to 500 mL of artificial seawater (salinity 30) to prepare the exposure solutions, with a solvent control group containing an equivalent volume of DMSO. Embryos were randomly divided into four groups (four replicates per group) and placed in Petri dishes containing 30 mL of exposure solution. Incubation was conducted in a climate-controlled chamber under 26 ± 1.0 °C and a 14 h:10 h light—dark cycle, with the exposure solution renewed every 24 h. Newly hatched larvae were transferred to new Petri dishes.

### 2.2. Embryonic Development Monitoring and Heart Rate Quantification

During the experiment, embryo mortality was recorded every 24 h. Embryonic hatching and developmental progression were observed under a stereomicroscope, with developmental delays and malformations photographed for documentation. On day 14 of exposure, the hatching rate (number of hatched eggs/total eggs) was calculated for each group. To quantify heart rates, embryos were video-recorded for 10 s under a stereomicroscope on days 6 (30 larvae per group) and 9 (20 larvae per group) of exposure, and heartbeats were counted from the recordings. Following hatching, larvae were fed boiled egg yolk solution twice daily for the first 3 days, then transitioned to twice-daily feedings of Artemia salina nauplii thereafter.

### 2.3. Measurement of Larval Survival, Body Length, and Heart Rate

On days 12 of exposure, marine medaka larvae were placed under a stereomicroscope to measure body length and heart rate. Following heart rate quantification, larvae were sampled (8 larvae per replicate, three replicates per group) and immediately preserved in 2 mL nuclease-free centrifuge tubes with 1 mL Trizol reagent for total RNA extraction. During this period, daily mortality was recorded to calculate larval survival rate.

### 2.4. Larval Swimming Behavior Analysis

Swimming behavior of marine medaka larvae was analyzed using the ZebraLab video–track system (ViewPoint, France). On days 12 of exposure, eight larvae per replicate were subjected to behavioral tests under both dark and light conditions. Prior to testing, larvae were acclimated in 24-well plates for 10 min, followed by a 20 min behavioral recording period. Data on swimming time, distance, and trajectory were automatically quantified every 120 s. Swimming activity was categorized as: high-speed swimming (>10 mm/s), medium-speed swimming (1–10 mm/s), and low-speed swimming or freezing (<1 mm/s). All experimental parameters were standardized across dark and light conditions.

### 2.5. Primer Design and Synthesis

Based on mRNA sequences obtained from our in-house transcriptome database and the NCBI database, specific qPCR primers targeting amplicons <250 bp were designed using Primer Premier 6.0 software (PREMIER Biosoft, USA). Primers were synthesized by Genewiz (Suzhou, China). Primer sequences are provided in Supplementary Table S1.

### 2.6. RNA Extraction and qPCR Analysis

Total RNA was extracted from medaka samples using Trizol reagent (Invitrogen, Carlsbad, CA, USA) following the manufacturer’s protocol. RNA integrity was assessed by 1% agarose gel electrophoresis (Sangon Biotech Ltd. Beijing, China), and purity (A260/A280 ratio) was measured using a NanoDrop 2000 spectrophotometer (Thermo Fisher Scientific, Waltham, MA, USA). All RNA samples exhibited A260/A280 ratios between 1.8 and 2.0, with distinct 18S and 28S rRNA bands confirming quality. Complementary DNA (cDNA) was synthesized from total RNA using the HiScript First–Strand cDNA Synthesis Kit (Novozymes, Nanjing, China) according to the manufacturer’s instructions. Quantitative real-time PCR (qPCR) was performed on a Roche LightCycler^®^ 96 System with a 15 μL reaction mixture containing: 7.5 μL 2× PerfectStart™ Green qPCR SuperMix (Tsingke Biotechnology, Guangzhou, China); 0.3 μL each of forward and reverse primers (10 μM); 2 μL cDNA template; 4.9 μL ddH_2_O. The thermal cycling conditions were 95 °C for 30 s (initial denaturation); 40 cycles of 95 °C for 5 s, 60 °C for 15 s, and 72 °C for 10 s (fluorescence acquisition); Melt curve analysis (65–95 °C, 0.5 °C/s increments). *actb2* and *gapdh* were used as reference genes for normalization. Relative gene expression was calculated using the 2^−ΔΔCt^ method, with results expressed as log_2_ fold-changes relative to the solvent control.

### 2.7. Statistical Analysis

All experimental data are presented as mean ± standard deviation (SD). One-way ANOVA followed by Dunnett’s post hoc test were performed to assess the statistical significance of differences between BPAF-treated groups and the solvent control group using GraphPad Prism 9 software (GraphPad Software, USA). Significance levels were denoted as: *p* < 0.05 (*), *p* < 0.01 (**), *p* < 0.001 (***), and *p* < 0.0001 (****).

### 2.8. Ethical Approval

All animal experiments were conducted in strict accordance with the Guide for the Care and Use of Laboratory Animals and approved by the Animal Research and Ethics Committee of Guangdong Ocean University (Approval No. 201903003).

## 3. Results

### 3.1. BPAF Exposure Impacts on Embryonic Development

**Figure 1:**
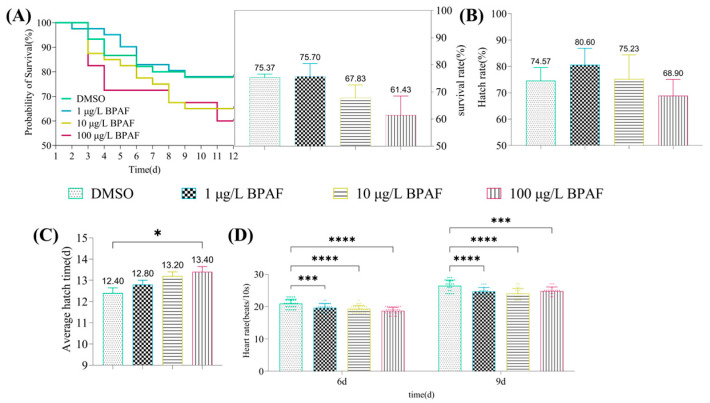
Effect of BPAF exposure on embryo-larval survival (**A**), hatching (**B**,**C**), and heart rate ((**D**), 6d N = 30; 9d N = 20) in marine medaka. Compared to the control, statistically signiffcant differences are shown with asterisks (* *p* ≤ 0.05, *** *p* ≤ 0.001, **** *p* ≤ 0.0001).

**Figure 2:**
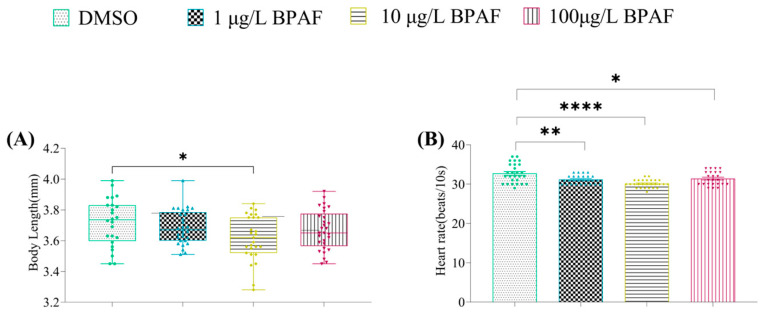
Body length ((**A**), N = 24), heart rate ((**B**), N = 24) of newly hatched marine medaka larvae. Compared to the control, statistically signiffcant differences are shown with asterisks (* *p* ≤ 0.05, ** *p* ≤ 0.01, **** *p* ≤ 0.0001).

**Figure 3:**
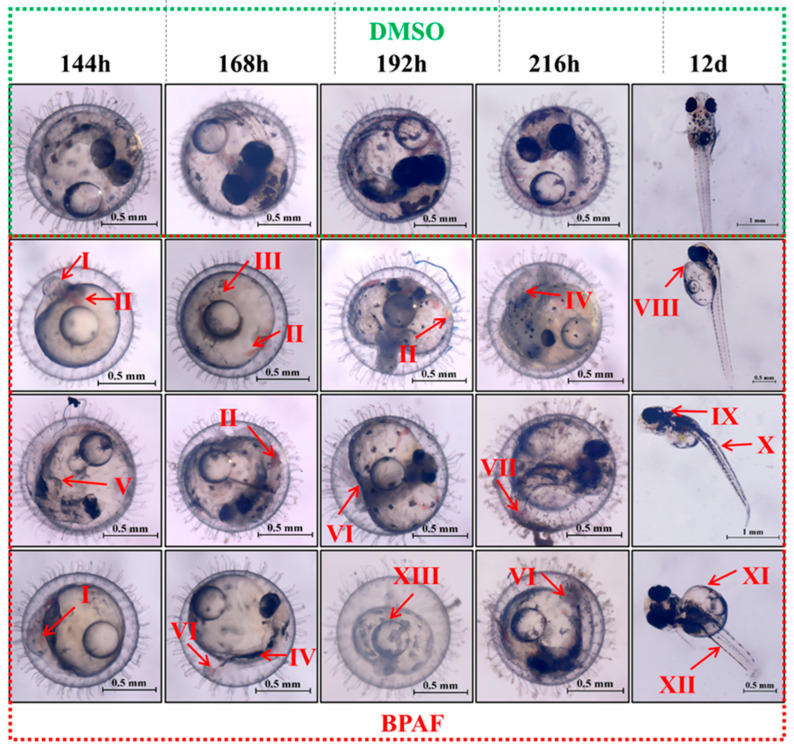
Effect of BPAF exposure on embryonic developmental morphology in marine medaka. I: eye dysplasia (lack of melanin in the eyes); II: cardiovascular bleeding; III: overall dysplasia; IV: truncal dysplasia and insufficient melanin pigmentation; V: overall deformity; VI: curved tail; VII: tail bleeding; VIII: cardiac edema; IX: craniofacial dysplasia; X: curvature of notochord; XI: yolk sac cysts; XII: short body length; XIII: abnormal yolk sac.

Overall, BPAF exposure exhibited no significant impact on the survival or hatching rates of marine medaka embryos. However, embryonic survival showed a downward trend with increasing BPAF concentrations, while hatching rates increased initially then decreased (Figure 1A–C). Notably, higher BPAF concentrations accelerated embryonic mortality onset and prolonged hatching duration, with 100 µg/L BPAF significantly delaying hatching time (Figure 1A–C). All BPAF concentrations significantly reduced embryonic heart rates on days 6 and 9 of exposure, demonstrating a concentration-dependent decline (Figure 1D). BPAF exposure had an inhibitory effect on the body length and heart rate of hatchlings, and the body length of medaka larvae in the 10 µg/L BPAF group was significantly lower than that of the control group (Figure 2A); the heart rate of medaka larvae was significantly reduced by 1 µg/L, 10 µg/L, and 100 µg/L BPAF, with the greatest effect on the heart rate of larval larvae being observed in the 10 µg/L BPAF group (Figure 2B). Additionally, BPAF exposure induced developmental delays and malformations in embryos, including underdeveloped eyes, cardiovascular hemorrhage, reduced melanin deposition, pericardial edema, and yolk sac abnormalities (Figure 3).

### 3.2. BPAF-Induced Alterations in Larval Swimming Behavior

**Figure 4:**
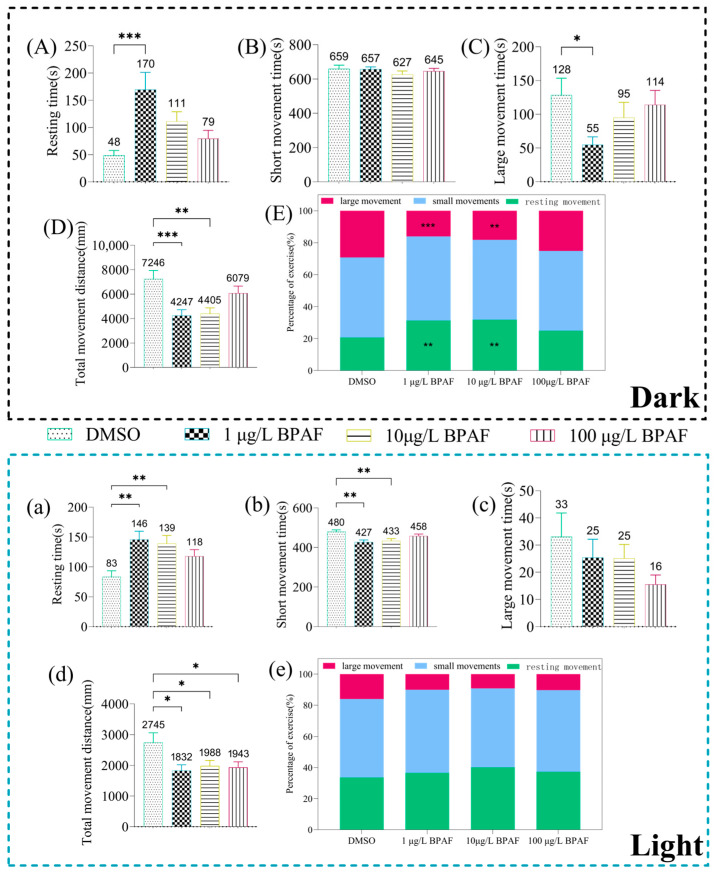
Swimming behavior of newly hatched marine medaka larvae in dark/light environments. (**A**,**a**): Resting states (N = 32). (**B**,**b**): Small movement states (N = 32). (**C**,**c**): Large movement states (N = 32). (**D**,**d**): Total movement distance (N = 32). (**E**,**e**): Percentage of movement by stage. Compared to the control, statistically signiffcant differences are shown with asterisks (* *p* ≤ 0.05, ** *p* ≤ 0.01, *** *p* ≤ 0.001).

BPAF exposure significantly impaired swimming behavior in newly hatched marine medaka larvae. Under dark conditions, both 1 and 10 µg/L BPAF reduced total movement distance (Figure 4D). Specifically, 1 µg/L BPAF increased resting time (Figure 4A) and decreased large movement time (Figure 4C). Furthermore, these concentrations elevated the proportion of resting episodes while reducing large movement episodes compared to the control (Figure 4a). Under light conditions, 1 µg/L BPAF, 10 µg/L BPAF, and 100 µg/L BPAF all significantly reduced the total movement distance of the larvae (Figure 4d), and compared with the control group, 1 µg/L BPAF and 10 µg/L BPAF significantly increased the time that the larvae were in the resting time (Figure 4a), decreased the time that the larvae were in the small movement time (Figure 4b); there was no significant difference between the groups in terms of type of movement (Figure 4e).

### 3.3. Effects of BPAF Exposure on Expression Levels of Cardiac Development-Related Genes in Marine Medaka Larvae

**Figure 5:**
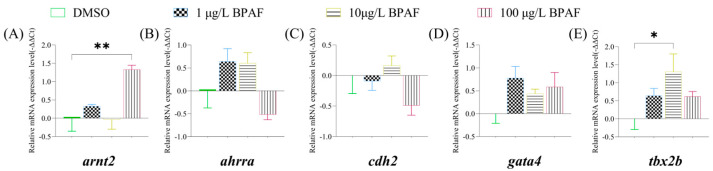
Analysis of gene expression levels related to cardiac development system of marine medaka larvae. (**A**): expression levels of *arnt2*; (**B**): expression levels of *ahrra*; (**C**): expression levels of *cdh2*; (**D**): expression levels of *gata4*; (**E**): expression levels of *tbx2b.* Compared to the control, statistically signiffcant differences are shown with asterisks (* *p* ≤ 0.05, ** *p* ≤ 0.01).

Based on the phenotype of BPAF-induced reduction in heart rate in newly hatched marine medaka larvae, this study evaluated the expression levels of cardiac development-related genes. In newly hatched larvae, exposure to 10 µg/L BPAF significantly increased the expression level of *tbx2b* compared to the control group (Figure 5E), while 100 µg/L BPAF exposure significantly upregulated *arnt2* expression (Figure 5A).

### 3.4. Effects of BPAF Exposure on Expression Levels of HPT Axis-Related Genes in Marine Medaka Larvae

**Figure 6:**
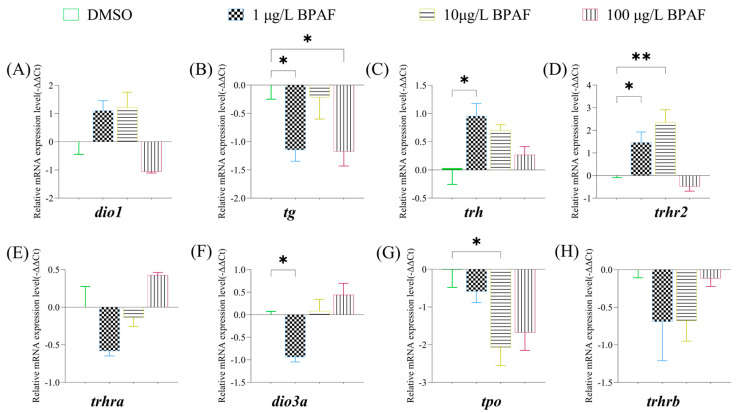
Analysis of gene expression levels associated with the HPT axis in marine medaka larvae. (**A**) expression levels of *dio1*; (**B**) expression levels of *tg*; (**C**) expression levels of *trh*; (**D**) expression levels of *trhr2*; (**E**) expression levels of *trhra.* (**F**) expression levels of *dio3a; (***G**) expression levels of *tpo*; (**H**) expression levels of *trhrb*; Compared to the control, statistically signiffcant differences are shown with asterisks (* *p* ≤ 0.05, ** *p* ≤ 0.01).

BPAF exposure disrupted the expression of HPT axis-related genes in marine medaka larvae. In newly hatched larvae, 1 µg/L BPAF significantly downregulated *tg* (Figure 6B) and *dio3a* (Figure 6F) while upregulating *trh* (Figure 6C) and *trhr2* (Figure 6D); 10 µg/L BPAF upregulated *trhr2* (Figure 6D) but downregulated *tpo* (Figure 6G); 100 µg/L BPAF selectively reduced *tg* expression (Figure 6B).

### 3.5. Effects of BPAF Exposure on Expression Levels of Nervous System-Related Genes in Marine Medaka Larvae

**Figure 7:**
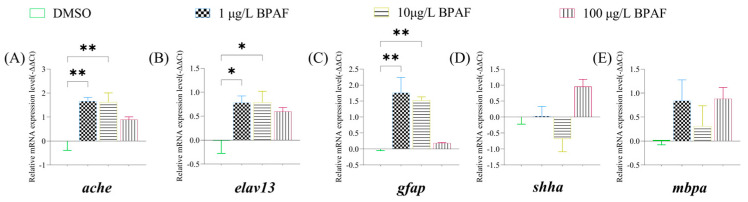
Analysis of the expression levels of nervous system-related genes in marine medaka larvae. (**A**) expression levels of *ache;* (**B**) expression levels of *elavl3*; (**C**) expression levels of *gfap*; (**D**) expression levels of *shha*; (**E**) expression levels of *mbpa.* Compared to the control, statistically signiffcant differences are shown with asterisks (* *p* ≤ 0.05, ** *p* ≤ 0.01).

Given the observed BPAF-induced hypoactivity in swimming behavior, this study assessed changes in expression levels of selected nervous system-related genes. In newly hatched larvae, *ache* expression was significantly elevated in the 1 µg/L and 10 µg/L BPAF groups compared to controls (Figure 7A); both 1 µg/L and 10 µg/L BPAF significantly upregulated *elavl3* (Figure 7B) and *gfap* (Figure 7C).

## 4. Discussion

### 4.1. Effects of BPAF Exposure on Embryonic Development, Survival Rate, and Hatching Rate in Marine Medaka

Fish embryos, as the initial and most vulnerable life stage, are highly sensitive to physicochemical environmental stressors, leading to reduced survival rates, impaired hatching success, and morphological malformations [25]. Investigating the effects of environmental pollutants on the morphological development of early life stages is critical. For example, exposure to 0.1 and 0.2 mg/L Cu^2+^ resulted in 100% mortality of *Gobiocypris rarus* embryos within 96 h [26], Mu et al. demonstrated that Cd^2+^, Hg^2+^, Cr^6+^, and Pb^2+^ exposure significantly reduced hatching rates and induced severe malformations in marine medaka embryos [27]. Sulfamethazine (SMZ) at 500–1000 mg/L significantly decreased zebrafish embryo survival, and concentrations above 0.001 mg/L caused malformations at 48 and 72 h post-fertilization (hpf) [28]. BPA exposure (5–10 µg/L) reduced spontaneous embryonic movement within 24 h and significantly lowered survival and hatching rates after 96 h [29]. Similarly, 2.5–3 mg/L BPAF induced developmental delays, pericardial edema, yolk sac edema, and death in zebrafish embryos [30], and Rao et al. reported that 500 μg/L BPAF suppressed hatching rates at 72 hpf, increased mortality at 72 and 96 hpf, reduced pigmentation at 50 and 500 μg/L, and caused pericardial edema, spinal curvature, and tail defects in larvae [31].

In this study, 100 μg/L BPAF exposure significantly prolonged embryonic hatching time, exhibiting a positive correlation with concentration, which aligns with previous findings that high-concentration BPAF suppresses hatching rates. Although no statistically significant differences in survival rates were observed across groups, a dose-dependent downward trend suggested potential lethal effects of BPAF on marine medaka embryos. During days 6–9 of exposure, BPAF-treated embryos exhibited developmental delays and malformations, including spinal curvature, yolk sac edema, and cardiovascular hemorrhage. Spinal curvature may relate to skeletal dysplasia or neurodevelopmental disruption, yolk sac edema likely results from BPAF-induced disruption of osmotic regulation, and cardiovascular hemorrhage could stem from abnormal development of the circulatory system. These findings are consistent with the malformation patterns reported by Huang [20]. 

### 4.2. Effects of BPAF Exposure on Growth, Survival, and HPT Axis-Related Gene Expression in Marine Medaka

The hypothalamic-pituitary-thyroid (HPT) axis is critically involved in vertebrate growth, organ differentiation, and regulation of thyroid hormone (THs) synthesis and metabolism [32]. In fish, embryonic and larval-juvenile development is predominantly governed by HPT axis regulation [33], with THs additionally playing pivotal roles in immune function, metabolism, and growth [34,35]. Thyrotropin-releasing hormone (Trh), synthesized in the hypothalamus, stimulates pituitary secretion of thyroid-stimulating hormone, which directs THs production in the thyroid gland [36]. Thyroglobulin (TG), secreted by thyroid follicular cells, is essential for triiodothyronine (T3) and thyroxine (T4) synthesis [37]. Deiodinase 1 (DIO 1) modulates THs activation and inactivation [38], while deiodinase 3a (DIO 3a) inactivates T4 and T3 via inner-ring deiodination, converting T4 to T3 or T3 to T2 [39]. Environmental contaminants perturb these pathways: Tang et al. [38] reported elevated *tg*, *dio1*, and *dio2* transcriptional levels in zebrafish larvae after 168 h exposure to 50 and 500 μg/L BPAF; 12.5 μg/L BPAF downregulated *dio1* and *dio3* in zebrafish juveniles after 7 days [40]; 50 μg/L Tetramethyl bisphenol F (TMBPF) suppressed *trh* and *trhr1* while upregulating *tpo* and *tg* in zebrafish after 14 days [41]; and 10–100 μg/L bisphenol F (BPF) increased *dio1*, *dio2*, *trh*, and *trhr1* expression in zebrafish embryos at 144 h [42]. Regarding estrogen receptor activation, BPAF clearly exhibits a dose-dependent agonist effect, particularly for Erα [43]. Although studies explicitly labeled as “non-monotonic” are lacking, evidence of diminished effects at high concentrations, biphasic competitive curves, and dual agonist/antagonist activity [44] strongly suggests a nonlinear dose-response relationship and the potential for non-monotonic effects. In this study, BPAF exposure altered expression of *tg*, *trh*, *trhr2*, *dio3a*, and *tpo* in marine medaka larvae, disrupting HPT axis-related gene expression and THs equilibrium, thereby inducing thyroid toxicity.

### 4.3. Effects of BPAF Exposure on Behavior and Nervous System-Related Gene Expression in Marine Medaka Larvae

Ethology serves as a robust tool for assessing chemical toxicity [45,46], with advances in digital imaging and AI-driven behavioral tracking now enabling quantitative analysis of subtle behavioral patterns [47]. Behavioral alterations in fish, which possess complex brain structures (telencephalon, diencephalon, midbrain, and hindbrain), can reflect internal physiological disruptions, making swimming behavior a sensitive toxicity endpoint [48]. In this study, both 1 and 10 μg/L BPAF exposure markedly inhibited locomotor activity in larvae. Similar behavioral suppression has been documented across bisphenol analogs: Gu Jie et al. reported that BPAF and BPAP exposure induced hypoactivity and freezing behavior in zebrafish larvae [49]; Yang et al. demonstrated BPA-induced reduction in movement under continuous light and light-dark stimuli in juvenile zebrafish [50]; and chronic 200 μg/L BPAF exposure over 70 days significantly decreased swimming distance in female marine medaka [51]. Studies indicate that BPAF exposure (at 50 µg/L and 500 µg/L) significantly increases anxiety-like behavior in adult zebrafish, accompanied by pathological changes in brain tissue and decreased acetylcholinesterase activity, demonstrating a dose-response relationship [31]. The behavioral outcomes of this study also exhibited a certain degree of dose-dependent effect, with behavioral suppression being more pronounced at lower concentrations. Additionally, this study observed consistently higher movement distances in dark versus light conditions across newly hatched, a phototactic response aligning with findings by Jennifer et al [52].

Acetylcholinesterase (ACHE) maintains normal neurotransmission by catalyzing acetylcholine hydrolysis and is a classical toxicological indicator reflecting pollutant-induced harm in aquatic organisms [53,54]. *shha* controls neural stem cell proliferation, neuron survival, and glial cell viability during animal development [55]. ELAVL3 and glial fibrillary acidic protein (GFAP) are key markers in neurodevelopment: ELAVL3 encodes a neural-specific RNA-binding protein, and its deficiency leads to severe neurodevelopmental defects and visual impairment [56], while GFAP, a cytoskeletal protein expressed in mature astrocytes of the central nervous system (CNS), participates in intercellular communication and cytoskeletal organization [57]. 0.05 and 0.5 mg/L BPAF exposure for 7 days significantly increased *gfap* expression in both male and female zebrafish while reducing *shha* levels in females [55]; 68.4 and 228 μg/L BPA exposure for 120 h upregulated *elavl3* and *gfap* in zebrafish embryos [58]; 3.0 mg/L BPS exposure for 6 days downregulated *α1-tubulin*, *elavl3*, *gap43*, *mbp*, *syn2a*, and *gfap*, impairing larval movement [59]; and Bi Sichao et al. observed dose-dependent increases in *ache* expression in planarians exposed to BPA [60]. Collectively, BPAF-induced neurotoxicity disrupts locomotor capacity in larvae, and the observed behavioral deficits further validate its neurotoxic effects.

### 4.4. Effects of BPAF Exposure on Heart Rate and Cardiovascular System-Related Gene Expression in Marine Medaka

*Tbx2b*, a critical regulator of early cardiac development, ensures proper chamber septation and cardiomyocyte differentiation by modulating chamber-specific gene expression [61]. The aryl hydrocarbon receptor (AHR) forms complexes with aryl hydrocarbon receptor nuclear translocator (ARNT), influencing cardiac morphogenesis and functional maturation through downstream gene regulation [62]. In this study, BPAF exposure significantly upregulated *arnt2* and *tbx2b* in newly hatched larvae. These results indicate that BPAF-induced dysregulation of cardiovascular genes (*arnt2*, *tbx2b*, *ahrra*) impairs cardiac development, aligning with the observed bradycardia in BPAF-treated larvae. Furthermore, cardiac dysfunction disrupts systemic circulation, potentially slowing nutrient delivery and causing developmental delays, behavioral deficits, or mortality [63], which corroborates the suppressed swimming activity and reduced survival rates in BPAF-exposed larvae.

## 5. Conclusions

In this 14-day exposure experiment, we comprehensively evaluated the toxic effects of BPAF on the embryonic to larval stages of marine medaka. It was found that, exposure to BPAF reduces the survival of marine medaka, and its lethality increases with increasing concentration. BPAF disrupts the expression of the HPT axis and genes related to heart development, affecting embryonic and juvenile growth and interfering with the normal regulation of heart rate. In addition, it alters the expression of genes related to the nervous system, thereby inhibiting the swimming behavior of juvenile fish.

## Data Availability

Data will be made available on request.

## Supplementary Materials

The following supporting information can be downloaded at: https://www.mdpi.com/article/10.3390/toxics13090773/s1, Table S1: qPCR primers and their sequences.

## References

[1] Zhang C., Wu X., Li S., Dou L., Zhou L., Wang F., Ma K., Huang D., Pan Y., Gu J. (2021). Perinatal low-dose bisphenol AF exposure impairs synaptic plasticity and cognitive function of adult offspring in a sex-dependent manner. Sci. Total Environ..

[2] Song S., Ruan T., Wang T., Liu R., Jiang G. (2012). Distribution and preliminary exposure assessment of bisphenol af (bpaf) in various environmental matrices around a manufacturing plant in China. Environ. Sci. Technol..

[3] Feng Y., Yin J., Jiao Z., Shi J., Li M., Shao B. (2012). Bisphenol AF may cause testosterone reduction by directlyaffecting testis function in adult male rats. Toxicol. Lett..

[4] Song S., Song M., Zeng L., Wang T., Liu R., Ruan T., Jiang G. (2014). Occurrence and profiles of bisphenol analogues in municipal sewage sludge in China. Environ. Pollut..

[5] Zhang S., Fan Y., Qian X., Wu Z., Feng S., Xu W., Wang G. (2024). Spatiotemporal distribution, source apportionment, and ecological risk of bisphenol analogues in a highly urbanized river basin. Sci. Total Environ..

[6] Yu X., Xue J., Yao H., Xue J.C., Yao H., Wu Q., Venkatesan A.K., Halden R.U., Kannan K. (2015). Occurrence and estrogenic potency of eight bisphenol analogs insewage sludge from the U.S. EPA targeted national sewage sludge survey. J. Hazard. Mater..

[7] Cunha S.C., Ferreira R., Marmelo I., Vieira L.R., Anacleto P., Maulvault A., Marques A., Guilhermino L., Fernandes J.O. (2022). Occurrence and seasonal variation of several endocrine disruptor compounds (pesticides, bisphenols, musks and UV-filters) in water and sediments from the estuaries of tagus and douro rivers (NE atlantic ocean coast). Sci. Total Environ..

[8] Gil-Solsona R., Castano-Ortiz J.M., Munoz-Mas R., Insa S., Farre M., Ospina-Alvarez N., Santos L.H.M.L.M., Garcia-Pimentel M., Barcelo D., Rodriguez-Mozaz S. (2022). A holistic assessment of the sources, prevalence, and distribution of bisphenol a and analogues in water, sediments, biota and plastic litter of the ebro delta (spain). Environ. Pollut..

[9] Choi Y.J., Lee L.S. (2017). Aerobic soil biodegradation of bisphenol (BPA) alternatives bisphenol S and bisphenol AF compared to BPA. Environ. Sci. Technol..

[10] Wiraagni I.A., Mohd M.A., Rashid R.A., Haron D.E.b.M. (2020). Trace level detection of bisphenol a analogues and parabens by LC-MS/MS in human plasma from malaysians. BioMed Res. Int..

[11] Wang Q., Cao Y., Zeng H., Liang Y., Ma J., Lu X. (2019). Ultrasound-enhanced zero-valent copper activation of persulfate for the degradation of bisphenol AF. Chem. Eng. J..

[12] Wallace C.W., Fordahl S.C. (2022). Obesity and dietary fat influence dopamine neurotransmission: Exploring the convergence of metabolic state, physiological stress, and inflammation on dopaminergic control of food intake. Nutr. Res. Rev..

[13] Tucker D.K., Bouknight S.H., Brar S.S., Kissling G.E., Fenton S.E. (2018). Evaluation of prenatal exposure to bisphenol analogues on development and long-term health of the mammary gland in female mice. Environ. Health Perspect..

[14] Zhu X. (2017). Neurotoxicity Effect of Bisphenol Exposure on Adult Zebrafish (*Danio rerio*). Master’s Thesis.

[15] Tang T. (2016). Disruption of the Thyroidal Axis Cognitive Ability in Zebrafish (*Danio rerio*) Exposed to BPAF. Ph.D. Thesis.

[16] Yang Y. (2016). Toxic Effects and Thyroid Disruption of Bisphenol AF on Zebrafish Embryos and Larvae. Master’s Thesis.

[17] Zhao X., Zhang Y., Yu T., Cai L., Liang J., Chen Z., Pan C., Yang M. (2023). Transcriptomics-based analysis of sex-differentiated mechanisms of hepatotoxicity in zebrafish after long-term exposure to bisphenol AF. Ecotoxicol. Environ. Saf..

[18] Shi J., Jiao Z., Zheng S., Li M., Zhang J., Feng Y., Yin J., Shao B. (2015). Long-term effects of bisphenol AF (BPAF) on hormonal balance and genes of hypothalamus-pituitary-gonad axis and liver of zebrafish (*Danio rerio*), and the impact on offspring. Chemosphere.

[19] Chen Y., Chen X., Li X., Liu Y., Guo Y., Wang Z., Dong Z. (2022). Effects of bisphenol AF on growth, behavior, histology and gene expression in marine medaka (*Oryzias melastigma*). Chemosphere.

[20] Huang Z., Gao J., Chen Y., Huan Z., Liu Y., Zhou T., Dong Z. (2023). Toxic effects of bisphenol AF on the embryonic development of marine medaka (*Oryzias melastigma*). Environ. Toxicol..

[21] Hong H., Shen R., Liu W., Li D., Huang L., Shi D. (2015). Developmental toxicity of three hexabromocyclododecane diastereoisomers in embryos of the marine medaka *Oryzias melastigma*. Mar. Pollut. Bull..

[22] Du M., Zhang D., Yan C., Zhang X. (2012). Developmental toxicity evaluation of three hexabromocyclododecane diastereoisomers on zebrafish embryos. Aquat. Toxicol..

[23] Chen Y., Wang X., Ran H. (2016). Developmental stages of a marine model fish–medaka *Oryzias melastigma*. Oceanol. Limnol. Sin..

[24] Wang Y., Liu H., Yu D. (2017). Observation of embryonic development of marine medaka (*Oryzias melastigma*). Mar. Sci..

[25] Herzig A., Winkler H. (2006). The influence of temperature on the embryonic development of three cyprinid fishes, abramis brama, chalcalburnus chalcoides mento and vimba vimba. J. Fish Biol..

[26] Shi J., Wang Y. (2014). Toxic Effects of Cu^2+^ on the Embryonic Development of *Gobiocypris rarus*. J. Neijiang Norm. Univ..

[27] Mu J., Wang Y., Wang X., Wang J. (2011). Toxic Effects of Cadmium, Mercury, Chromium and Lead on the Early Life Stage of Marine Medaka (*Oryzias melastigma*). Asian J. Ecotoxicol..

[28] Liu L., Lyn P., Yan Y. (2018). Acute toxicities of sulfamethazine to zebrafish embryos. Chin. Fish. Qual. Stand..

[29] Han J. (2023). Study of the Effects of Bisphenol A (BPA) on the Development of Zebrafish Embryos. Master’s Thesis.

[30] Yang Y., Tang T.-L., Chen Y.-W., Tang W.-H., Yang F. (2020). The role of chorion around embryos in toxic effects of bisphenol AF exposure on embryonic zebrafish (*Danio rerio*) development. Estuar. Coast. Shelf Sci..

[31] Rao C., Cao X., Li L., Zhou J., Sun D., Li B., Guo S., Yuan R., Cui H., Chen J. (2022). Bisphenol AF induces multiple behavioral and biochemical changes in zebrafish (*Danio rerio*) at different life stages. Aquat. Toxicol..

[32] Kloas W., Lutz I. (2006). Amphibians as model to study endocrine disrupters. J. Chromatogr. A.

[33] Jugan M.-L., Levi Y., Blondeau J.-P. (2010). Endocrine disruptors and thyroid hormone physiology. Biochem. Pharmacol..

[34] Liu Y.W., Chan W.K. (2002). Thyroid hormones are important for embryonic to larval transitory phase in zebrafish. Differentiation.

[35] Power D.M., Llewellyn L., Faustino M., Nowell M.A., Björnsson B.T., Einarsdottir I.E., Canario A.V.M., Sweeney G.E. (2001). Thyroid hormones in growth and development of fish. Comp. Biochem. Physiol. C Toxicol. Pharmacol..

[36] Iwamuro S., Yamada M., Kato M., Kikuyama S. (2006). Effects of bisphenol A on thyroid hormone-dependent up-regulation of thyroid hormone receptor α and β down-regulation of retinoid X receptor γ in xenopus tail culture. Life Sci..

[37] Jin Y., Liu H., Han Z., Hua X. (2018). Effects of BDE-28 and BDE-99 on Functional Gene Expression along HPT, HPG and HPA Axes during Early Life Stages of Zebrafish. Asian J. Ecotoxicol..

[38] Tang T., Yang Y., Chen Y., Tang W., Wang F., Diao X. (2015). Thyroid disruption in zebrafish larvae by short-term exposure to bisphenol AF. Int. J. Environ. Res. Public Health.

[39] Bianco A.C., Salvatore D., Gereben B., Berry M.J., Larsen P.R. (2002). Biochemistry, cellular and molecular biology, and physiological roles of the iodothyronine selenodeiodinases. Endocr. Rev..

[40] Chen P., Wang R., Chen G., An B., Liu M., Wang Q., Tao Y. (2022). Thyroid endocrine disruption and hepatotoxicity induced by bisphenol AF: Integrated zebrafish embryotoxicity test and deep learning. Sci. Total Environ..

[41] Kim H., Ji K. (2022). Effects of tetramethyl bisphenol F on thyroid and growth hormone-related endocrine systems in zebrafish larvae. Ecotoxicol. Environ. Saf..

[42] Yang Q., Liu J., Ding J., Chen L. (2021). Endocrine Disrupting Effects of Bisphenol F on Early Life Stages of Zebrafish. Asian J. Ecotoxicol..

[43] Kitamura S., Suzuki T., Sanoh S., Kohta R., Jinno N., Sugihara K., Yoshihara S., Fujimoto N., Watanabe H., Ohta S. (2005). Comparative study of the endocrine-disrupting activity of bisphenol a and 19 related compounds. Toxicol. Sci..

[44] Iwamoto M., Masuya T., Hosose M., Tagawa K., Ishibashi T., Suyama K., Nose T., Yoshihara E., Downes M., Evans R.M. (2021). Bisphenol a derivatives act as novel coactivator-binding inhibitors for estrogen receptor β. J. Biol. Chem..

[45] Peng Y., Wei Y., Ding Y., Duan J. (2017). Development of drug toxicity and novel strategy for toxicity of Chinese materiamedica based on zebrafish model. Chin. Tradit. Herb. Drugs.

[46] Wu M. (2021). Endocrine Disrupting Effects of Bisphenol F on Early Life Stages of Zebrafish. Master’s Thesis.

[47] Dell A.I., Bender J.A., Branson K., Couzin I.D., de Polavieja G.G., Noldus L.P.J.J., Pérez-Escudero A., Perona P., Straw A.D., Wikelski M. (2014). Automated image-based tracking and its application in ecology. Trends Ecol. Evol..

[48] Kou G. (2021). Estrogen-like Compounds Developmental Toxicity and Influence Onendocrine Axis in Early Life of Zebrafish. Master’s Thesis.

[49] Gu J., Wang H., Liao Z., Shi L., Ji G. (2019). Neurodevelopmental toxicities of bisphenol AP and bisphenol AF in early life of zebrafish. J. Environ. Occup. Med..

[50] Yang L., Shi Q., Zhou B. (2017). The Effects of BPA on Neurobehavior and Neurotransmitters of Larval Zebrafish (*Danio rerio*). Asian J. Ecotoxicol..

[51] Li X. (2022). Toxic Effects of Bisphenols in Marine Medaka and the Effect of Salinity on the Toxic Effects of Bisphenol A. Master’s Thesis.

[52] Fitzgerald J.A., Kirla K.T., Zinner C.P., vom Berg C.M. (2019). Emergence of consistent intra-individual locomotor patterns during zebrafish development. Sci. Rep..

[53] Li S., Zhang J., Jiang M., Wu H., Liu L., Ruan H. (2014). Effects of ivermectin on the physiological andbiochemical characteristic features of Daniorerio. J. Saf. Environ..

[54] Yue X., Yang A., Xu P., Hu X., Zhu H., Bao X. (2019). Effect of Antimony on the Enzyme Activity of *Danio rerio*. Biotechnol. Bull..

[55] Zhu X., Tang T., Peng X., Tang W. (2017). Bisphenol AF Exposure Reduces Learning and Memory Ability and Influences Expression of Nervous System Genes in Zebrafish. Asian J. Ecotoxicol..

[56] Kalwy S.A., Smith R. (1994). Mechanisms of myelin basic protein and proteolipid protein targeting in oligodendrocytes (review). Mol. Membr. Biol..

[57] Nielsen A.L., Jorgensen A.L. (2003). Structural and functional characterization of the zebrafish gene for glial fibrillary acidic protein, GFAP. Gene.

[58] Kim S.S., Hwang K.-S., Yang J.Y., Chae J.S., Kim G.R., Kan H., Jung M.H., Lee H.-Y., Song J.S., Ahn S. (2020). Neurochemical and behavioral analysis by acute exposure to bisphenol a in zebrafish larvae model. Chemosphere.

[59] Gu J., Zhang J., Chen Y., Wang H., Guo M., Wang L., Wang Z., Wu S., Shi L., Gu A. (2019). Neurobehavioral effects of bisphenol S exposure in early life stages of zebrafish larvae (*Danio rerio*). Chemosphere.

[60] Bi S., Wu S., Pang Q., Zhao T., Xue M., Zhang X. (2019). Effects of BPA Exposure on Acute Toxicity and Neurological Enzymes in *Dugesia japonica*. Genom. Appl. Biol..

[61] Christoffels V.M., Hoogaars W.M.H., Tessari A., Clout D.E.W., Moorman A.F.M., Campione M. (2004). T-box transcription factor Tbx2 represses differentiation and formation of the cardiac chambers. Dev. Dyn..

[62] Antkiewicz D.S., Peterson R.E., Heideman W. (2006). Blocking expression of AHR2 and ARNT1 in zebrafish larvae protects against cardiac toxicity of 2,3,7,8-tetrachlorodibenzo-p-dioxin. Toxicol. Sci..

[63] Kim K.-H., Antkiewicz D.S., Yan L., Eliceiri K.W., Heideman W., Peterson R.E., Lee Y. (2007). Lrrc10 is required for early heart development and function in zebrafish. Dev. Biol..

