# Supplementary material for: Toxic Effects of BPAF on Marine Medaka (Oryzias melastigma) During Embryo–Larval Stages
Source: Toxics. 2025 Sep 12;13(9):773. doi: 10.3390/toxics13090773 (PMC12473650; doi:10.3390/toxics13090773)
Supplement: Supplementary file 1 [file toxics-13-00773-s001.zip › toxics-3840636-supplementary.pdf]

Table S1. qPCR primers and their sequences.

Gene	Primer sequence (3`-5`)	Product size (bp)	Accession number
actb2	F: GCAGGTCATCACCATCGGCAAT<br>R:CCTCCAGACAGCACAGTGTTGG	171	XM_024272788.2
gapdh	F:GTGGTGCCAGCCAGAACATC<br>R:TTCCAGGCGGACTGTGAGG	152	XM 024267865.2
arnt2	F:GCATACAAGCCCTCCTTCCTAAC;<br>R:TCCGACACATAAATCACCCG	116	XM_024281904.1
ahrra	F:TAAACGGAGGGATGGGTGAC;<br>R:GTGATTGTCTTTGCCGACGG	113	XM_036217415.1
cdh2	F:CCAAAGTGTTCCCTCACGAAG;<br>R:CCGCTGATTCTGGTTATGGTC	169	XM_024273520.2
gata4	F:CAGTGACACCGAGCAGCA;<br>R:ACACGCTGTGGTTCTCCG	90	XM_024291891.2
tbx2b	F:TCCTTTGCTCCATGCAGACC;<br>R:TTGTCGATCTTCAGCTGCGT	187	XM_024258183.2
dio1	F:TCATCTACATCACGGAGGCG;<br>R:CATTTCATCAACGACCACGG	123	XM_024278314.2
tg	F:GGCTCTCTGCTCTGTGGTTG;<br>R:GCCCGACTTCTCCTTGTTGTAG	122	XM_024269321.2
trh	F:GCGAAGATGAAATCCACTCC;<br>R:CAGAAATGTGTCCTGCCAAG	81	XM_024292087.2
trhr2	F:TGTGGAGTGGGAATCATTGG;<br>R:AGGCTCACCAGGTAACAGTTGG	100	XM_024295484.2
trhra	F: TGTCTGAGTGGAGAGAATGAGAGG<br>R: GGTTTGGTTGTGGAGCATC	150	XM_024264169.2
dio3a	F: AAGGTCGTTTATCAAGGTGGC<br>R: CAGCATCTTTGTTTCTGTCATAGC	162	XM_024278595.2
tpo	F:ACTGTGTGGTTCTCCAACCC<br>R: GCTTCCTTGCCAAACAGCAT	197	XM_036210000.1
trhrb	F: CATCACAGCCTTCACCATTG<br>R: ACAGGTAGAACCACATTACGCAG	146	XM_024260826.2
ache	F:GAGAGTCCAGATCTGAGGGGA;<br>R:TGAAGGTCAGAAGTGTCGGC	101	XM_024288220.2
elavl3	F: CGCAGCCGAACCCATAAC<br>R: TGAAGGGGTCCCGTGTAG	114	XM_024272587.2
gfap	F: GCGGAGCAGAAGCAGGTCACTA<br>R: CGACGACGAGAGGACGACTGTT	197	XM_024296052.1
shha	F:TGGGTTCGACTGGGTCTACT;<br>R:ATCGTGGAGGATCCTGGGAA	111	XM_024279618.2
mbpa	F: GCGGAGCAGAAGCAGGTCACTA<br>R: CGACGACGAGAGGACGACTGTT	197	XM_024296052.1
